# Serum neurofilament light chain levels are correlated with the infarct volume in patients with acute ischemic stroke

**DOI:** 10.1097/MD.0000000000030849

**Published:** 2022-09-30

**Authors:** June Woo Ahn, Jaechun Hwang, Myunghoon Lee, Jae Hyoung Kim, Hee-Jin Cho, Ho-Won Lee, Mi-Yeon Eun

**Affiliations:** a Department of Neurology, Kyungpook National University Chilgok Hospital, School of Medicine, Kyungpook National University, Daegu, South Korea; b Research Center, D&P Biotech, Inc., Daegu, South Korea; c Brain Science and Engineering Institute, Kyungpook National University, Daegu, South Korea.

**Keywords:** Asian, infarct volume, neurofilament light chain, stroke

## Abstract

Neurofilament light chains (NfLs) are promising biomarkers of neuroaxonal damage in stroke patients. We investigated the correlations between NfL levels and infarct volume, initial stroke severity, and functional outcomes at discharge in patients with acute ischemic stroke.

We prospectively included 15 patients with first-ever acute ischemic stroke and 8 age- and sex-matched healthy controls without other neurological disorders. Serum NfL levels were measured using the single-molecule array (Simoa) technique twice within 24 hours of admission (NfL_1D_) and on the seventh hospital day (NfL_7D_) in patients with stroke and once in healthy controls. We assessed the infarct volume on diffusion-weighted magnetic resonance imaging using the free software ITK-SNAP.

Serum NfL_1D_ levels in stroke patients were significantly higher (28.4 pg/mL; interquartile range [IQR], 43.0) than in healthy controls (14.5 pg/mL; IQR, 3.2; *P* = .005). Temporal pattern analyses demonstrated that NfL_7D_ levels were increased (114.0 pg/mL; IQR, 109.6) compared to NfL_1D_ levels in all stroke patients (*P* = .001). There was a strong correlation between NfL_7D_ levels and infarct volume (*R* = 0.67, *P* = .007). The difference between NfL_1D_ and NfL_7D_ (NfL_diff_ levels) was strongly correlated with the infarct volume (*R* = 0.63; *P* = .013). However, there was no statistically significant correlation between NfL levels and the initial stroke severity or functional outcomes at discharge.

NfL levels in the subacute stage of stroke and the NfL difference between admission and 7th day of hospital were correlated with infarct volume in patients with acute ischemic stroke.

## 1. Introduction

Neurofilaments (Nf) are specific neuronal cytoskeletal proteins consisting of four subunits: Nf light, Nf medium, Nf heavy chains, and alpha-internexin. Neurofilament light chains (NfLs) are the most abundant type 4 intermediate filaments in the neuroaxonal compartment. When the axons of the central nervous system are damaged, NfL is released into the extracellular space, increasing NfL levels not only in the cerebrospinal fluid (CSF) but also in the peripheral blood.^[1]^ This suggests that serum NfL may act as a biomarker of neuroaxonal damage in a variety of neurological disorders, including neurodegenerative diseases, multiple sclerosis, amyotrophic lateral sclerosis, and traumatic brain injury.^[2–6]^

Ischemic stroke causes neuroaxonal damage following cerebral artery occlusion. The extent of neuroaxonal injury due to ischemia may be related to stroke lesion volume. In addition, the larger the lesion volume, the higher is the probability of severe symptoms, and poor clinical outcomes. Therefore, serum NfL may be a promising biomarker for predicting stroke severity and outcome. Several recent studies have reported that NfL is associated with severity of symptoms, infarct size, and prognosis of acute ischemic stroke.^[7–12]^ However, this remains controversial, and relevant studies in Asian patients are scarce.

We aimed to evaluate the difference in serum NfL levels between patients with acute ischemic stroke and controls, and the association of serum NfL levels with infarct volume, stroke severity, and functional outcome at discharge in Asian patients with acute ischemic stroke.

## 2. Materials and Methods

### 2.1. Study design and participants

This was a single-center prospective observational study. Considering the limitations of experimental resources, we planned to include 15 patients with acute ischemic stroke and 8 healthy controls. We consecutively recruited patients with acute ischemic stroke who were admitted to the neurology department between April 2020 and July 2020. The eligibility criteria were as follows: age ≥ 20 years and acute ischemic stroke within 7 days of symptom onset. Acute ischemic stroke was defined as an episode of focal neurological deficits lasting ≥24 hours with a relevant lesion on brain magnetic resonance (MR) images. Patients with a history of stroke or other neurological disorders that may affect serum NfL levels (Alzheimer disease, Parkinson disease, multiple sclerosis, amyotrophic lateral sclerosis, or traumatic brain injury) were excluded from the study.^[1]^ And those who did not provide consent to participate in the study were also excluded. Blood sample processing for NfL testing was performed during daytime on weekdays. If the collected blood samples could not be processed immediately after sampling, patients were excluded from the study. We also recruited eight healthy controls matched for age and sex in an outpatient clinic without other neurological disorders during the same period.

### 2.2. Study protocol approvals and patient consents

This study was approved by the Institutional Review Board of Kyungpook National University Chilgok Hospital. Written informed consent was obtained from all participants or their guardians.

### 2.3. Data acquisition

We collected demographic data and vascular risk factors, including hypertension, diabetes mellitus, dyslipidemia, coronary artery disease, atrial fibrillation, and current smoking status, from the medical records. Laboratory findings, brain imaging findings, and stroke parameters were also determined. Stroke severity was assessed using the National Institutes of Health Stroke Scale (NIHSS) on 1st and 7th hospital days. We evaluated functional status at discharge using the modified Rankin Scale (mRS).

### 2.4. Blood sampling and serum NfL measurement

Peripheral venous blood (8 mL) for measuring NfL levels was collected twice within 24 hour of admission (NfL_1D_) and on the 7th hospital day (7 ± 1 days; NfL_7D_) from patients with acute ischemic stroke. Blood samples were collected from age-and sex-matched healthy controls (HCs). Blood samples were immediately placed in gel separator tubes and maintained at room temperature for 30 to 60 minutes. After centrifugation for 10 minutes at 2000 × g at room temperature, the serum was aliquoted and stored at −80 °C.

Serum NfL concentrations were measured using a Simoa NF-light Advantage Kit on a 4th generation single-molecule array (Simoa) HD-X Analyzer (Quanterix, MA, USA). Diluted calibrators and serum samples were analyzed in duplicate, and intra-assay coefficients of variation (CVs) below 15% were considered appropriate.

### 2.5. Infarct volume estimation

Diffusion-weighted magnetic resonance imaging (MRI) was performed on the day of admission in all patients. All MRI scans, except one, were performed using 3T MRI. The slice thickness of the diffusion-weighted MRI ranged from 2 to 5 mm. The lesions on diffusion MR images were manually segmented, and the infarct volume was calculated using ITK-SNAP 3.6.0.^[13]^

### 2.6. Statistical analysis

Continuous variables are presented as medians with interquartile ranges (IQR), and categorical variables are expressed as numbers with percentages. Baseline characteristics were compared using Fisher exact test for categorical variables and Mann–Whitney U-test for continuous variables. The Wilcoxon signed-rank test was used to compare NfL_1D_ and NfL_7D_ levels. NfL_diff_ was defined as the difference between NfL_7D_ and NfL_1D_. Finally, Spearman rank correlation coefficient was used to measure the association between serum NfL levels and infarct volume, stroke severity, and functional outcomes at discharge. A two-sided *P* < .05 was considered statistically significant. Data were analyzed using the statistical software R version 4.0.5.

### 2.7. Data access statement

The datasets generated and/or analyzed during the current study are not publicly available but are available from the corresponding author upon reasonable request.

## 3. Results

**Figure 1.**
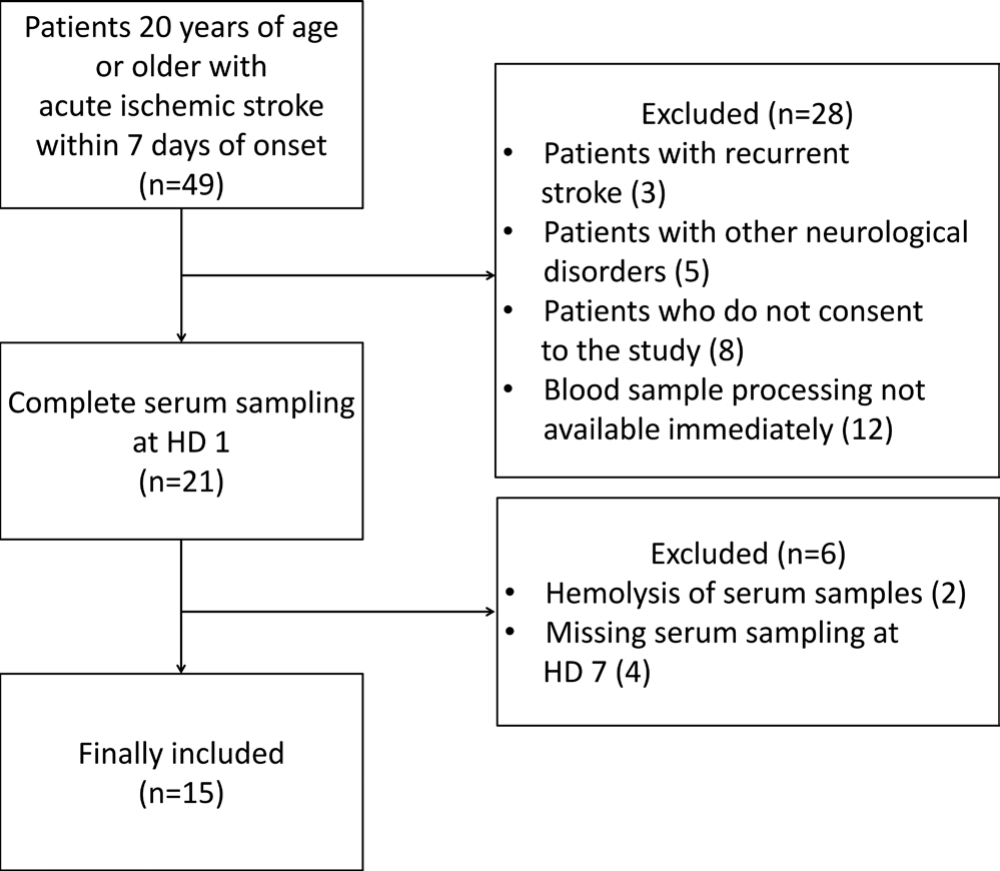
Selection of patient population with acute ischemic stroke.

Between April and July 2020, 66 patients with cerebral infarction visited our hospital. A total of 49 patients met the inclusion criteria. Eight patients were excluded because they had a history of stroke or other neurological disorders such as Alzheimer disease or Parkinson disease. Eight patients did not consent to participate in this study. Twelve patients were excluded because their blood samples could not be immediately processed for NfL testing. Initially, 21 patients were enrolled in this study. However, two patients dropped out due to hemolysis of blood samples, and four patients dropped out because of missing a second sample on the 7th hospital day. Finally, 15 patients with acute ischemic stroke and 8 age-and sex-matched healthy controls were included in this study (Fig. 1).

The baseline characteristics of the patients with ischemic stroke and healthy controls are presented in Table 1. Most of the study participants were male (78.3%), and the median age of each group was 72 years. The rate of dyslipidemia tended to be higher in the patient group, but the difference was not statistically significant. The median time from symptom onset to admission was 8 hours, and the median initial NIHSS score was 5. The etiologies of stroke in 15 patients according to the TOAST classification were large-artery atherosclerosis in 5 patients, small vessel occlusion in 4 patients, cardioembolism in 3 patients, other determined etiology in 1 patient, and undetermined etiology in 2 patients. The median time from symptom onset to admission was 9.6 hours (IQR, 32.1).

**Table 1:** Baseline characteristics of patients with ischemic stroke and healthy controls.

Variables	Patients (n = 15)	Controls (n = 8)	*P* *
Patient demographics			
Age	72.0 (18.0)	72.0 (9.3)	0.974
Male (%)	12 (75.0)	6 (80.0)	1.000
Body mass index	23.5 (5.5)	25.9 (5.4)	0.220
Medical history			
Hypertension	10 (66.7)	7 (87.5)	0.369
Diabetes mellitus	5 (33.3)	5 (62.5)	0.221
Dyslipidemia	9 (60.0)	1 (12.5)	0.074
Current smoking	5 (33.3)	1 (12.5)	0.369
Coronary artery disease	1 (6.7)	0 (0)	1.000
Atrial fibrillation	0 (0)	0 (0)	N/A
Time from symptom onset to admission (h)	9.6 (32.1)	N/A	N/A
Initial NIHSS score	5.0 (4.0)	N/A	N/A
NIHSS score on the seventh HD	3.0 (3.0)	N/A	N/A
Discharge mRS	2.0 (3.0)	N/A	N/A

Categorical variables are represented as numbers (%) and continuous variables as medians (IQR).

* Continuous variables were compared between the groups using the Mann–Whitney *U* test. Fisher exact test was used to analyze categorical variables.

HD = hospital day; IQR = interquartile range, NIHSS = National Institutes of Health Stroke Scale, mRS = modified rankin scale, N/A = not available.

**Figure 2.**
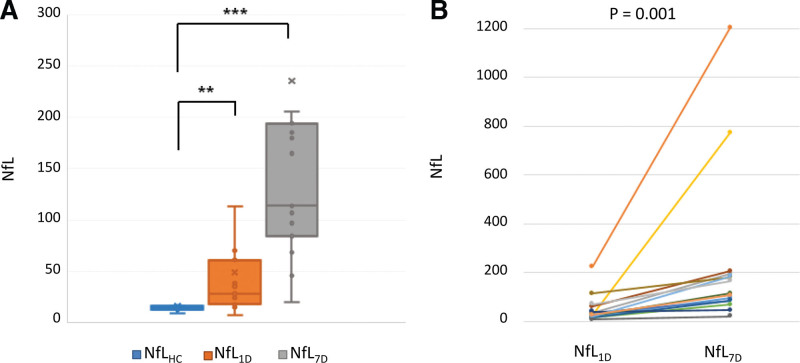
Serum NfL levels in the study population. (A) NfL levels in healthy controls and patients with acute ischemic stroke (HD 1 and HD 7) using the Mann–Whitney U-test. (B) Temporal pattern of NfL levels in every patient using the Wilcoxon signed-rank test. HD = hospital day, NfL = neurofilament light chain, NfL_HC_ = neurofilament light chain in healthy controls, NfL_1D_ = neurofilament light chain within 24 h of admission in patients, NfL_7D_ = neurofilament light chain on the seventh hospital day.

The first blood samples for baseline NfL levels were collected within 24 hour of admission from every patient. The second blood sampling for NfL_7D_ levels was conducted at a median of seven days (IQR, 1). The CVs for each duplicate sample was <15%. In healthy controls, NfL levels tended to increase with increasing age, but the difference was not statistically significant (*R* = 0.55, *P* = .157). Baseline NfL levels were significantly higher in patients (28.4 pg/mL; IQR, 43.0) than in healthy controls (14.5 pg/mL; IQR, 3.2; *P* = .005). Temporal pattern analyses in patients with stroke demonstrated that NfL_7D_ levels were increased (114.0 pg/mL; IQR, 109.6) compared to baseline NfL in all stroke patients (*P* = .001) (Table 2, Fig. 2).

**Table 2:** Serum neurofilament light chain levels and infarct volume in patients with acute ischemic stroke and controls.

Variables	Patients (n = 15)	Controls (n = 8)	*P* *
NfL_1D_ (pg/mL)	28.4 (43.0)	14.5 (3.2)	0.005
NfL_7D_ (pg/mL)	114.0 (109.6)	N/A	N/A
NfL_diff_	80.3 (91.95)	N/A	N/A
Infarct volume (mL)	12 (75.0)	N/A	N/A

Continuous variables were presented as median (IQR).

* Continuous variables were compared between the groups using the Mann–Whitney *U* test.

HD = hospital day, NfL_1D_ = neurofilament light chain within 24 h of admission in patients, NfL_7D_ = neurofilament light chain on the seventh hospital day, N/A = not available.

**Figure 3.**
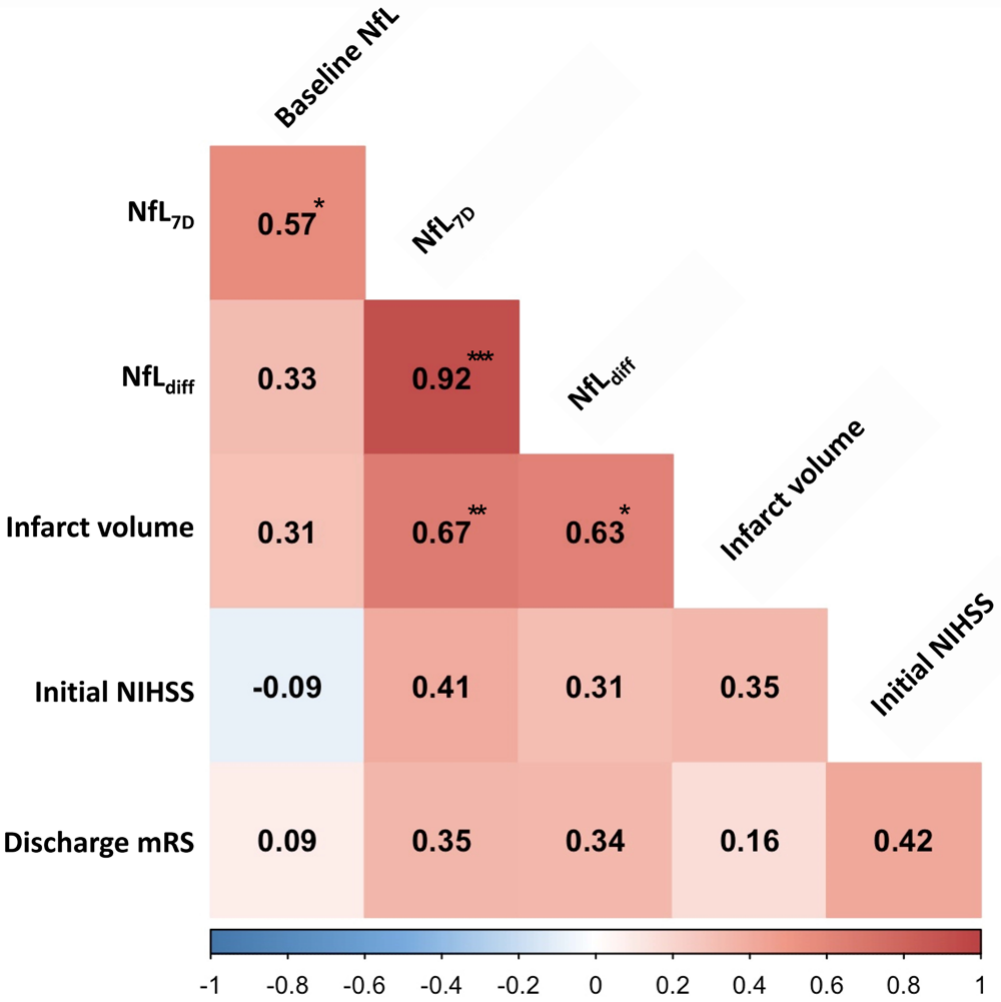
Correlation between serum NfL levels and infarct volume, initial NIHSS score, and discharge mRS. Analysis was conducted using Spearman rank correlation analysis. * means *P* < .05; ** means *P* < .01; *** means *P* < .001. NfL = neurofilament light chain, NIHSS = National Institutes of Health Stroke Scale, mRS = modified rankin scale.

We conducted correlation analyses between serum NfL levels and stroke severity, infarct volume, and functional outcomes at discharge (Fig. 3). The NfL_7D_ levels were moderately correlated with the NfL_1D_ levels (*R* = 0.57; *P* = .026). There was a strong correlation between NfL_7D_ levels and infarct volume (*R* = 0.67, *P* = .007). The NfL_diff_ levels were strongly correlated with infarct volume (*R* = 0.63; *P* = .013). However, there was no statistically significant correlation between NfL_7D_ (*R* = 0.41; *P* = .131) or NfL_diff_ (*R* = 0.31; *P* = .261) and the initial stroke severity. With regard to the functional outcome at discharge using the mRS, there was no correlation with the NfL levels.

## 4. Discussion

Our study demonstrated that serum NfL level might be a promising biomarker of neuroaxonal injury after ischemic stroke. Serum NfL levels were higher in patients with acute ischemic stroke than those in age-and sex-matched healthy controls. Serum NfL levels increase over time in patients with acute stroke. Serum NfL_7D_ and NfL_diff_ levels strongly correlated with infarct volume.

This is the first study in South Korea to evaluate serum NfL levels using the Simoa method in patients with acute ischemic stroke. Although several studies have reported that NfL levels are higher in patients with acute stroke than in healthy controls or patients with transient ischemic attack, there have been few reports in Asia.^[7,10,11,14]^ Our study identified increased NfL levels in Asian patients with stroke and demonstrated the diagnostic value of NfL.

Temporal pattern analysis demonstrated that NfL levels increased on the 7th hospital day compared to admission in all patients. Previous studies have reported that serum NfL levels increase over time up to two weeks after acute ischemic stroke and are maintained for 3 to 6 months.^[9,10,15,16]^ Cerebral arterial occlusion induces neuronal necrosis followed by secondary injury via immune and inflammatory processes.^[17]^ Our results suggest that both acute and subsequent neuroaxonal injury after acute ischemic stroke may contribute to increased NfL_7D_ levels after ischemic stroke. Based on this assumption, NfL levels might be helpful in assessing the treatment response to neuroprotective therapy, as well as reperfusion therapy, and further research is warranted.

Our results demonstrated that NfL_7D_ levels correlated with infarct volume. However, there was no correlation between the NfL_1D_ levels and infarction volume. Previous studies have reported controversial results. In a study by Marchis et al, serum NfL levels collected within 24 hour of symptom onset in patients with acute ischemic stroke and infarct size on admission diffusion MRI did not show an association.^[7]^ On the other hand, in a study by Juha Onatsu et al, there was an association between infarct volume measured with a computerized tomography 2 days after the onset of symptoms and randomly collected serum NfL levels at 1 to 12 days.^[8]^ In another recent study, serum NfL_7D_ levels, but not NfL_1D_ levels, correlated with infarct volumes, as in our study, and the correlation coefficient (*R* = 0.74) was similar to our result (*R* = 0.66).^[10]^ Based on these results, including ours, subacute stage NfL levels seemed to better reflect neuroaxonal damage due to ischemic stroke rather than early NfL levels.

Interestingly, we further investigated the relationship between NfL_diff_ and infarct volume, and found a strong correlation between them. NfL levels increase with age and in diverse neurological diseases, such as amyotrophic lateral sclerosis, multiple sclerosis, and Alzheimer dementia.^[1]^ Thus, we excluded patients with other neurological disorders. As NfL_diff_ in each individual might cancel out the factors that could affect NfL levels, NfL_diff_ could be used to predict neuroaxonal damage in stroke patients of different ages or with other neurological disorders.

Although NfL_7D_ levels tended to increase as the initial NIHSS score increased, we did not find a significant correlation between serum NfL levels and stroke severity based on the NIHSS score on admission. Conversely, several studies have reported an association between NfL levels and stroke severity.^[7,11,15]^ This difference may be attributed to the lack of statistical power owing to the small number of participants in this study. However, stroke severity is not always correlated with tissue damage. Even a small infarction in the eloquent area can cause severe neurological deficits. Furthermore, in the case of the NIHSS system, stroke severity tended to be overestimated in left hemispheric lesions. Therefore, serum NfL and NIHSS scores may be less correlated.

In this study, we used the Simoa platform, which is the most sensitive tool for measuring the NfL.^[18]^ The test results were reliable for all the duplicate samples. Neurofilaments confer structural stability to neurons and NfL is the most abundant and soluble subunit of neurofilaments.^[1]^ NfL is constantly released in small amounts even under normal physiological conditions. However, neuroaxonal damage caused by cerebral vessel occlusion can cause a dramatic increase in CSF NfL levels, with some NfL leaking into the blood. Unlike previous methods, such as ELISA, the Simoa method can accurately measure even low concentrations of NfL and shows a high correlation between CSF and blood.^[18]^ Therefore, serum NfL measurement using the Simoa method can be a useful noninvasive biomarker for ischemic stroke, especially in cases that cannot undergo MRI or have negative results on MRI.

Our study had several limitations. First, the number of patients and samples enrolled in the study was small because of the limitations of experimental resources. Therefore, we conducted correlation analysis without adjusting for age or other confounding factors. Second, we could not evaluate the association between NfL level and long-term outcomes in patients with ischemic stroke. Furthermore, although we registered patients consecutively, there may have been a selection bias, as patients who visited during the daytime were included owing to the limitations of the experimental conditions. Finally, since this study was conducted only in Koreans, there is a limitation in the generalization. However, a study of Caucasians reported similar results.

## 5. Conclusion

This study demonstrated that NfL levels in the subacute stage of stroke and the NfL difference between admission and 7th day of hospital were correlated with infarct volume in patients with acute ischemic stroke. Despite several limitations, our study is meaningful as it is the first in Korea to study the relationship between stroke and serum NfL levels using the Simoa technique, which is currently known to be the most sensitive to NfL measurement. Further research with a larger patient population and long-term follow-up is warranted to confirm our study results.

## Author contributions

**Conceptualization:** Jaechun Hwang, Ho-Won Lee, Mi-Yeon Eun.

**Data curation:** June Woo Ahn, Mi-Yeon Eun.

**Formal analysis:** Mi-Yeon Eun.

**Funding acquisition:** Ho-Won Lee.

**Investigation:** June Woo Ahn, Jaechun Hwang, Myunghoon Lee, Jae Hyoung Kim, Hee-Jin Cho.

**Methodology:** Myunghoon Lee.

**Supervision:** Mi-Yeon Eun.

**Writing – original draft:** June Woo Ahn.

**Writing – review & editing:** Ho-Won Lee, Mi-Yeon Eun.
